# Living in Constant Crisis—When Stress Management Becomes the Problem

**DOI:** 10.1371/journal.pbio.1001999

**Published:** 2014-11-18

**Authors:** Roland G. Roberts

**Affiliations:** Public Library of Science, Cambridge, United Kingdom

Emergency services are supposed to just kick in during times of need, sort out the problem, and then return to base. But what happens if the emergency period never ends—is there a downside to living in a constant state of crisis? Our bodies have many surveillance systems; most of the time they stand idly by and only spring into action when the need arises, but we're starting to appreciate that their inappropriate or long-term activation may not be a good thing.

One such crisis is the presence in a cell of misfolded proteins—either because proteins have failed to fold properly or because some catastrophe (such as a rise in temperature) has caused previously folded proteins to unfold and misfold. If things go wrong with a protein's folding, the consequences can be severe, including sticking to unrelated proteins and compromising their function and sticking to other copies of themselves to form large, insoluble, and potentially toxic aggregates.

The process of managing folding is known collectively as proteostasis. Under normal circumstances, the cell contains the right number and type of chaperones (protein-folding helpers) and other agents to fold the proteins that are continually being made and to refold the occasional folding error. However, when a crisis occurs and the cell detects unfolded or misfolded proteins, it activates the appropriate proteostatic stress pathway, resulting in the production of an army of chaperones that try to refold the casualties and minimise any harm done. The stress response pathways also include a disposal option whereby those proteins that can't be rescued are tagged with ubiquitin and carried away for degradation. Once the crisis is over and the number of unfolded or misfolded proteins returns to manageable levels, all systems go back to their basal “business as usual” mode. Job done.

But what happens if levels of misfolded proteins are *always* high? This occurs in genetic diseases where the effect of the mutation disrupts the normal folding of the encoded protein. Clearly this could result in chronic (life-long) activation of the proteostatic stress response. On the whole, the stress response is assumed to be a good thing, and indeed, some have suggested that artificially triggering this response could be useful therapeutically.

However, an article by Daniela Martino Roth, William Balch, and colleagues, just published in *PLOS Biology*, suggests that chronic activation of the stress response can be detrimental, exacerbating the disease phenotype. They study the mutations involved in four human genetic diseases where protein folding is compromised—three in which the misfolding causes disease by reducing the function of the protein in question (cystic fibrosis, Niemann-Pick disease, and α1-antitrypsin deficiency) and one where the misfolding causes aggregation that is itself pathogenic (Alzheimer's disease).

There were already reports of possible disadvantages of chronic stress response activation, but this paper clarifies the situation. The study concentrates initially on F508del-CFTR, a very common cystic fibrosis mutation. The amino acid missing from this mutant protein isn't directly needed for the function of the CFTR protein, but it is needed for its correct folding and trafficking to the plasma membrane. Instead of reaching its destination and acting as a chloride channel, the misfolded mutant protein becomes trapped elsewhere and tagged for degradation.

This trapping was known to depend on the chaperones Hsp70 and Hsp90, which are up-regulated by the main cytoplasmic stress pathway, the heat shock response (HSR). Working with bronchial epithelial cells from cystic fibrosis patients, the authors checked whether triggering the HSR (by heat-shocking the cells) would exacerbate the mutant CFTR trapping. It did; 90% of the mutant CFTR protein was rapidly degraded while the regular protein functioned as normal. Overexpression of a constitutively active form of HSF1, the transcription factor that drives the HSR, had the same result, showing that this was down to the HSR rather than the heat used to trigger it.

This suggested that rather than always helping, the proteostatic stress response could also hinder CFTR folding. The authors found that the phosphorylation state of HSF1 (a mark of activation) in CFTR mutant cells was elevated above normal levels. Levels of active trimeric HSF1 and of several of its target chaperones were also increased in the cystic fibrosis cells. This HSR activation was abolished if they silenced the mutant *CFTR* gene with RNA interference. The misfolding of the mutant CFTR protein therefore seems to induce a stress response that, in turn, makes the trapping and degradation worse, not better—the degree of induction was about 50% of that following full-blown heat shock. This chronic HSR activation also adversely affected the folding of other proteins in the same cell. The authors suggest that the misfolding mutant is triggering what they term a “maladaptive stress response.”

Just how general is this effect? The authors extended their study to several other genetic diseases in which misfolding is implicated, examining cells expressing an aggregating form of α1-antitrypsin or a mistrafficked form of NPC1 (responsible for α1-antitrypsin deficiency and one subtype of a lysosomal storage disorder, Niemann-Pick disease, respectively). Both mutant cell types showed signs of a similar maladaptive stress response, and depleting HSF1 improved disease-relevant protein states in each case. Similarly, two Alzheimer's models—one in worms and one in mice—also had up-regulated HSR, and HSF1 depletion reduced the paralysis seen in the worms.

**Figure 1:**
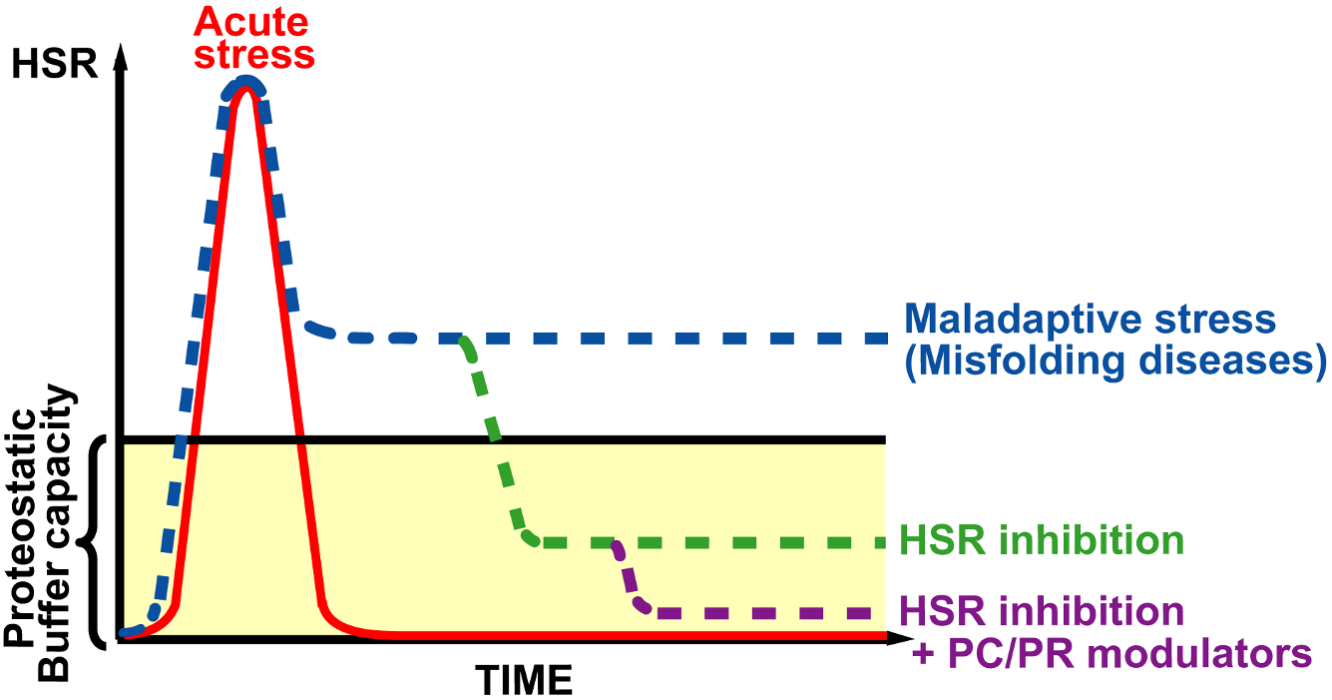
In the chronic presence of misfolding mutations, the normal acute HSR (red) can become maladaptive (blue). This can be rescued by inhibiting the HSR (green), and further aided (purple) by combining with drugs such as pharmacologic chaperones (PCs), which help to stabilise the protein fold or proteostasis regulators (PRs) that improve the folding environment. The overall effect is to enhance function of the disease-related misfolded protein.

So, if a maladaptive stress response can exacerbate the effects of disease mutations, what can we do about it? The authors demonstrated that breaking the stress response loop (by depleting HSF1 or P23, a protein that helps to activate it, or inhibiting HSF1) reduced the chronic activation of HSF1 in cystic fibrosis cells, increased the levels, stability, and trafficking of mutant CFTR protein, and, crucially, increased its chloride channel activity in the membrane—the very activity whose absence is responsible for the disease symptoms. Combining these treatments with VX809, a “corrector” drug that improves F508del-CFTR function, yields an even more pronounced positive effect (Figure 1).

The provocative message is that although acute activation of proteostatic stress responses is generally helpful, the long-term activation that is triggered by congenital misfolding mutations can be maladaptive, exacerbating the mutation's effects and feeding back into a vicious circle of chronic stress. So rather than promoting stress responses as a potential solution, we should also ask ourselves whether they are part of the problem. Stop the klaxon, and the state of emergency ends, allowing normal business to proceed.

**Roth DM, Hutt DM, Tong J, Bouchecareilh M, Wang N, et al. (2014) Modulation of the Maladaptive Stress Response to Manage Diseases of Protein Folding.** doi:10.1371/journal. pbio.1001998

